# End-of-the-year Review: Pediatric and Adult Congenital Heart Disease in 2025

**DOI:** 10.19102/icrm.2026.17017

**Published:** 2026-01-15

**Authors:** Johannes C. von Alvensleben

**Affiliations:** 1 Section of Electrophysiology, Division of Cardiology, Department of Pediatrics, Children’s Hospital Colorado, University of Colorado School of Medicine, Aurora, CO, USA

**Keywords:** Arrhythmias, congenital heart disease, pacemakers, pediatrics

In 2025, contemporary advances were observed in catheter ablation, arrhythmia management, and cardiac implantable electronic devices (CIEDs) in pediatric and adult patients with congenital heart disease (CHD). As is typical in this anatomically complex population, much of the data arise from single-center case series or limited patient cohorts. A national French registry reported by Waldman et al.^1^ on catheter ablation in CHD was the primary multicenter study from this year.

## Catheter ablation and arrhythmia management in congenital heart disease: an overview of the evidence

These studies collectively demonstrate that catheter ablation in CHD patients is feasible and generally safe, though recurrence rates remain substantial, particularly in complex substrates.^1–3^ Advanced mapping technologies and novel ablation techniques show promise, but the anatomic complexity of CHD continues to pose challenges for long-term arrhythmia control.^2–4^ Waldman and colleagues presented a French nationwide prospective registry of 1135 catheter ablation procedures in 998 CHD patients (mean age, 46 years) across 28 centers from 2020–2024, which demonstrated acute success in 94.4%, with rates exceeding 90% for all arrhythmias except ventricular arrhythmias (86.7%).^1^ The most common arrhythmias targeted were atrial flutter/tachycardia (59.6%), atrial fibrillation (AF; 17.2%), and ventricular arrhythmia (16.6%), with acute complications occurring in only 3.8% of procedures, including one death (0.1%). Freedom from arrhythmia recurrence was 77.3% at 1 year and 68.4% at 2 years, with significant variations based on arrhythmia type and underlying CHD complexity. The study concluded that catheter ablation in CHD demonstrates highly favorable acute outcomes with low complication rates, although recurrence rates vary depending on the targeted arrhythmia and underlying substrate.

### Ventricular arrhythmias in pediatric arrhythmogenic right ventricular cardiomyopathy

Radiofrequency catheter ablation for ventricular tachycardia (VT) in pediatric arrhythmogenic right ventricular cardiomyopathy (ARVC) demonstrates high recurrence rates despite initial success. In a study by Alahwany et al.^2^ of 15 pediatric patients (mean age, 15.5 years at symptom onset), electroanatomic mapping revealed universal basal right ventricular (RV) epicardial substrate involvement, with 54% showing endocardial involvement. While catheter ablation was safe, with no acute complications, 80% of patients required repeat ablations over 16.4 months of follow-up. VT-free survival reached 73% after multiple procedures (median, 2), although 27% required bilateral cardiac sympathetic denervation for recurrent arrhythmias. Notably, 13% progressed to heart failure, requiring transplantation within two decades of presentation.

### Postoperative arrhythmias

Early postoperative electrophysiology studies (EPS) and ablation can effectively reduce arrhythmia burden in CHD patients, although recurrence remains common. Among 50 patients undergoing EPS within 12 months of CHD surgery (representing only 0.2% of 28,902 operations), study authors Kerr et al.^3^ found that the most common mechanism was intra-atrial re-entrant tachycardia (46%), followed by ectopic atrial tachycardia (EAT) (26%). Acute procedural success was achieved in 82% of patients, with complications reported in 8%. Despite 54% experiencing arrhythmia recurrence during follow-up, the overall arrhythmia burden was significantly reduced.

EAT following CHD surgery typically resolves but carries a 13% risk of late recurrence. Laird-Gion et al.^4^ report that, among 167 patients developing EAT within 45 days of surgery (median age, 1.7 months; median onset, 7 days postoperatively), one-third experienced recurrence during initial hospitalization. While 56% were discharged on anti-arrhythmics for a median of 6 months, only 2% ultimately required EPS and ablation. Risk factors for late recurrence (>3 months) included prior arrhythmia, preoperative extracorporeal membrane oxygenation, specific surgical procedures (truncus arteriosus repair, bidirectional Glenn, coronary intervention), sustained EAT, intravenous anti-arrhythmic use, and early recurrence.

### Novel ablation strategies

Very high-power short-duration (vHPSD) ablation appears safe and effective for AF in adult CHD patients, particularly those with paroxysmal AF. In 66 adult CHD patients undergoing vHPSD ablation (70 W for 5–7 s or 60 W for 7–10 s), Lengauer et al.^5^ determined that no technique-related adverse events occurred, although vascular access complications affected 10.6%. At 1 year of follow-up, freedom from atrial arrhythmias off anti-arrhythmic drugs was 77.8% for patients with paroxysmal AF but only 43.6% for those with persistent AF. The mean procedure time was 123.6 min, with a radiofrequency ablation time of just 18.19 min, suggesting improved procedural efficiency.

### Arrhythmia localization and mapping

Standard electrocardiogram-based prediction algorithms have limited utility for focal atrial tachycardia (FAT) localization in adult CHD patients. An analysis by O’Connell et al.^6^ of 30 FATs in 29 adult CHD patients revealed that most originated from sites common to structurally normal hearts (particularly the crista terminalis, n = 14) rather than CHD-specific foci. While a negative or biphasic P-wave in V1 had 100% specificity for right atrial origin, other P-wave morphologies showed poor predictive value.

High-density electroanatomic mapping systems enable successful ablation of complex atrial arrhythmias in CHD, although recurrence rates remain significant. In 16 adult CHD patients undergoing 21 arrhythmia mapping procedures, Pravda et al.^7^ identified 21 intra-atrial re-entrant tachycardias and nine FATs. Procedural success was achieved in 66.6%, with partial success seen in 23.8% and no major adverse events. During 15.3 months of follow-up, 44% experienced recurrent arrhythmias requiring four redo procedures, although 18.8% were able to discontinue anti-arrhythmic medications.

## Clinical implications

The evidence supports several important clinical considerations:

1. Procedural success rates are high (82%–94% acute success across studies), but long-term recurrence remains common (13%–54% depending on substrate and arrhythmia type).^1–4^
2. Multiple procedures are often necessary, particularly for ventricular arrhythmias in ARVC and complex atrial substrates.^1,4^
3. Novel technologies, including vHPSD ablation and high-density mapping systems, may improve procedural efficiency and safety.^3,4^
4. Anatomic complexity significantly impacts outcomes, with lower success rates in patients with complex surgical atrial anatomy.^2,4^
5. Despite recurrence, overall arrhythmia burden is typically reduced following ablation, improving quality of life even when a complete cure is not achieved.^2,4^

These findings align with the consensus guidelines from the Pediatric and Congenital Electrophysiology Society and Heart Rhythm Society, which emphasize that catheter ablation in CHD patients requires specialized expertise, advanced mapping capabilities, and realistic expectations regarding the need for repeat procedures.

## Cardiac implantable electronic devices: an overview of the evidence

Pediatric and CHD patients require specialized approaches to CIED therapy that differ substantially from those of adult populations.^8^ Last year, Mah and Triedman published a comprehensive review of these devices in pediatrics and CHD. Key considerations include body size and growth, anticipated lifespan spanning decades, complex anatomy affecting implantation, and age-dependent rhythm pathologies.

### Epicardial lead performance

Epicardial leads remain essential for small children and those with a complex anatomy, although they carry higher complication rates than transvenous leads. Dechert et al.^9^ found in their recent study that early complications (<30 days) occur in approximately 20% of epicardial implantable cardioverter-defibrillator (ICD) cases versus 0% for transvenous cases. However, when adjusted for patient age and weight at implantation, long-term lead-dysfunction rates become comparable between epicardial and transvenous systems. Pleural coils demonstrate superior outcomes compared to epicardial coils in pediatric populations (hazard ratio, 0.38; 95% confidence interval, 0.15–0.96).

### Implantable cardioverter-defibrillator benefits and risks

In a German retrospective study by Fetcu et al.,^10^ 214 pediatric and young adult ICD recipients (median age, 23 years; median follow-up, 5.7 years) with CHD (61%), primary electrical disease (22%), or pediatric cardiomyopathy (17%) from 2001–2023 were examined. Appropriate therapy occurred in 41% overall, with significantly higher rates reported in secondary versus primary prevention recipients (56% vs. 26% at 5 years; *P* = .003), while complications were substantial: 36% required unplanned surgeries predominantly for lead-related issues, with abdominal generator placement and epicardial/extracardiac leads identified as specific risk factors, and inappropriate shocks occurred in 13%, including more frequently in CHD patients. These findings underscore that, while ICDs provide critical protection against sudden cardiac death in this population, the substantial complication burden necessitates meticulous patient selection and device strategy planning.

Combined subcutaneous ICD (S-ICD) and pacemaker devices offer solutions for complex CHD patients who require both defibrillation and pacing capabilities while avoiding transvenous leads. Sarubbi and colleagues^11^ examined 11 consecutive CHD patients who received S-ICDs after previously having pacemakers implanted, demonstrating this approach as a safe alternative to upgrading to transvenous ICD systems. All patients demonstrated good device compliance with no complications, including no infections or skin erosions during follow-up. This is particularly notable given that combined S-ICD and pacemaker systems have been associated with higher complication risks in other studies.

### Pacemaker device experience and cardiac resynchronization therapy

In their study, Papaccioli et al.^12^ examined 71 adults with moderate and complex CHD (mean age, 38.6 years) who received transvenous pacemakers, finding that fewer implanted leads correlated with fewer complications. In the dual-chamber group, 10 re-interventions were required for lead dysfunction (8 cases) and lead-related infective endocarditis (2 cases), while the single-lead group experienced only three lead dysfunctions requiring two re-interventions and no endocarditis. The authors emphasized that device selection should be individualized, balancing the hemodynamic benefits of dual-chamber pacing against increased complication risks from additional leads.

Cortez^13^ reported the first dual-chamber Aveir™ leadless pacemaker implantation (Abbott, Chicago, IL, USA) via the right internal jugular vein in a 13-year-old patient with congenital complete heart block who presented with presyncope and an average heart rate of <50 bpm. At 3-month follow-up, both atrial and ventricular parameters remained stable. This case demonstrates the feasibility of dual-chamber leadless pacing via internal jugular access in pediatric patients with CHD, representing an important advancement given that leadless pacemakers avoid pocket- and lead-related complications that are particularly problematic in young CHD patients who face decades of device dependency.

In a single-center retrospective study examining 45 adults with CHD (mean age, 54 ± 14 years) who received cardiac resynchronization therapy (CRT) between 2015 and 2022, Schamroth and colleagues^14^ presented average follow-up data spanning 5.2 ± 0.5 years. Their study cohort included 26 patients (57.8%) with a systemic left ventricle and 19 (42.2%) with a systemic RV. CRT demonstrated high efficacy regardless of baseline ejection fraction (EF) or ventricular morphology: among patients with reduced EF at baseline (<50%), 69.0% achieved a therapeutic response (defined as a ≥5% increase in systemic ventricular EF), while 92.3% of patients with preserved EF (≥50%) maintained their EF (preventative efficacy). Importantly, there was no significant difference in response rates between the systemic left ventricle and systemic RV groups (*P* = .15) or between baseline EF groups (*P* = .10). However, complications occurred in 28.9% of patients, underscoring the need for careful patient selection despite the overall favorable efficacy profile.

## Knowledge gaps and future directions

Despite the newest research, critical knowledge gaps persist in several areas: optimal timing of pacemaker implantation after postoperative atrioventricular block, risk factors for pacemaker-induced cardiomyopathy, optimal age and body size for transvenous lead implantation, and safety of magnetic resonance imaging with abandoned or epicardial leads. Multicenter prospective registries and high-quality retrospective data are essential to provide real-world evidence for new and existing CIED technologies in pediatric and CHD populations.
